# Up to 19-year cryopreservation does not impair hematopoietic progenitor function in cord blood: a 1129-unit analysis from the largest Korean public cord blood bank

**DOI:** 10.1093/stcltm/szag075

**Published:** 2026-08-25

**Authors:** Eun Youn Roh, Hye Ryun Lee, Sue Shin, Jong Hyun Yoon, Nam-Hee Kim, Hyun-Woong Park, Byoung Jae Kim

**Affiliations:** Department of Laboratory Medicine, SMG-SNU Boramae Medical Center, Seoul, 07061, Republic of Korea; Seoul Metropolitan Government Public Cord Blood Bank (ALLCORD), Seoul, 07061, Republic of Korea; Department of Laboratory Medicine, Seoul National University College of Medicine, Seoul, 03080, Republic of Korea; Department of Laboratory Medicine, National Medical Center, Seoul, 04564, Republic of Korea; Department of Laboratory Medicine, SMG-SNU Boramae Medical Center, Seoul, 07061, Republic of Korea; Seoul Metropolitan Government Public Cord Blood Bank (ALLCORD), Seoul, 07061, Republic of Korea; Department of Laboratory Medicine, Seoul National University College of Medicine, Seoul, 03080, Republic of Korea; Department of Laboratory Medicine, SMG-SNU Boramae Medical Center, Seoul, 07061, Republic of Korea; Seoul Metropolitan Government Public Cord Blood Bank (ALLCORD), Seoul, 07061, Republic of Korea; Department of Laboratory Medicine, Seoul National University College of Medicine, Seoul, 03080, Republic of Korea; Department of Laboratory Medicine, SMG-SNU Boramae Medical Center, Seoul, 07061, Republic of Korea; Department of Laboratory Medicine, Seoul National University College of Medicine, Seoul, 03080, Republic of Korea; Department of Laboratory Medicine, SMG-SNU Boramae Medical Center, Seoul, 07061, Republic of Korea; Department of Laboratory Medicine, Seoul National University College of Medicine, Seoul, 03080, Republic of Korea; Seoul Metropolitan Government Public Cord Blood Bank (ALLCORD), Seoul, 07061, Republic of Korea; Department of Obstetrics and Gynecology, SMG-SNU Boramae Medical Center, Seoul, 07061, Republic of Korea

**Keywords:** cord blood, cryopreservation, colony-forming units, hematopoietic stem cell, cellular therapy

## Abstract

**Background:**

Whether umbilical cord blood (UCB) quality is maintained during decades of cryopreservation is critical for public banking policy. We analyzed post-thaw quality of 1129 UCB units cryopreserved for up to 19 years at Korea’s largest public cord blood bank and examined implications for shelf-life policy.

**Methods:**

UCB units manually volume-reduced and cryopreserved between 2006 and 2025 underwent pre-transplant testing using their attached integral segments (median storage 6.5 years; range 16 days-19.0 years). Associations between storage duration and 19 quality indicators and 13 paired pre-/post-thaw complete blood count parameters were analyzed by Spearman correlation (*ρ*) before and after adjusting for era effects (processing and testing dates) to separate genuine storage effects from longitudinal analytical variation. Predictors of colony-forming unit (CFU) were ranked by univariate correlation and a Random Forest machine-learning model. Compliance with German/FACT-NetCord pre-transplant criteria was assessed.

**Results:**

Associations between storage duration and quality indicators were modest before adjustment (|*ρ*| ≤ 0.4) and were largely abolished once differences in testing era were taken into account: the storage-duration correlations for viable CD45+ and viable CD34+ both lost significance, as did CFU/CD34 + (*P* = .327). The post-thaw CD34+ count was the strongest predictor of CFU. Testing date, although ranked only seventh by simple correlation, rose to second in the Random Forest model, indicating an era effect not captured by pairwise analysis. The composite pre-transplant criteria pass rate was 96.2%, remaining above 93% across all storage bins.

**Conclusions:**

After up to 19 years of cryopreservation, per-CD34+ colony-forming capacity is preserved. A test-based shelf-life policy is more evidence-based than a predetermined expiration limit.

## Graphical Abstract

**Graphical Abstract:**
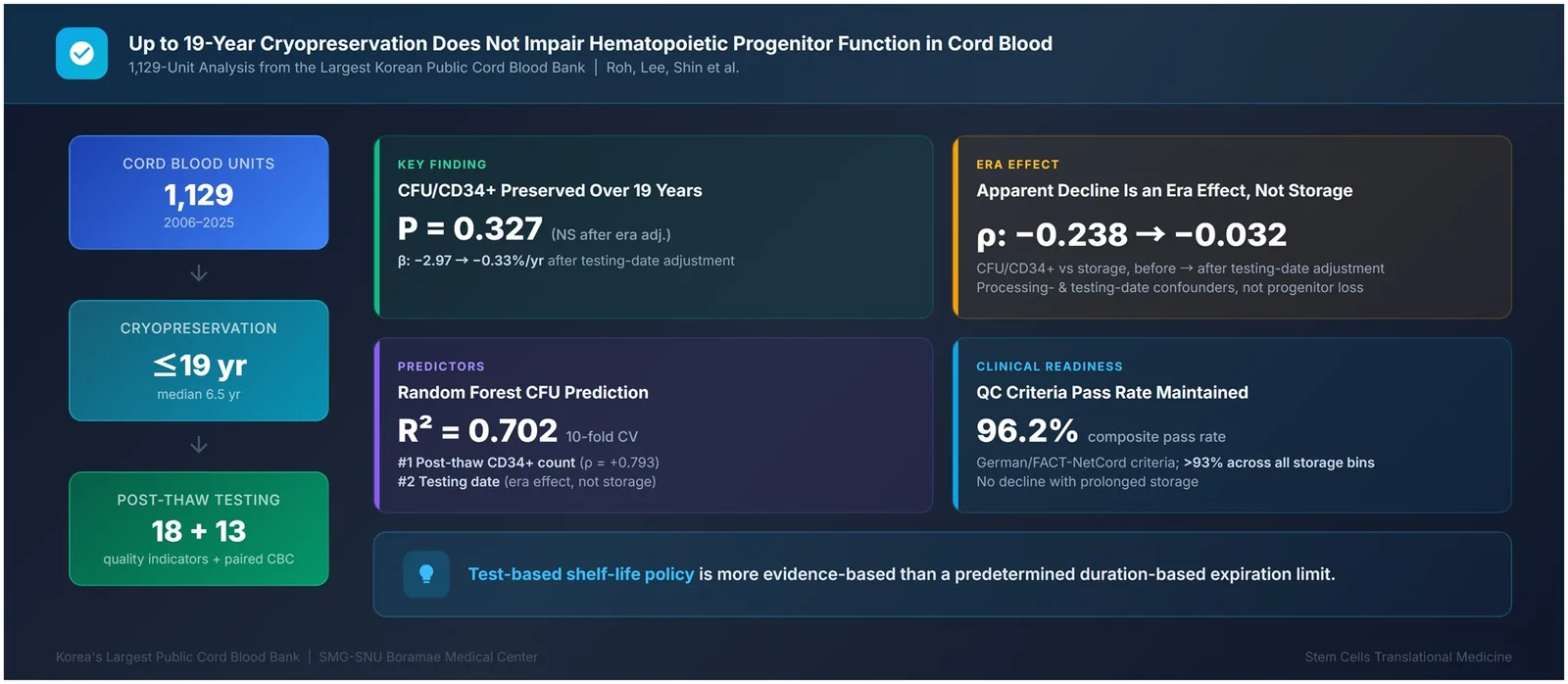

Significance Statement Cord blood banking relies on the assumption that frozen stem cells remain functional over time, yet few large-scale studies have examined quality after prolonged storage. This study demonstrates that the colony-forming capacity of stem cells is preserved after nearly two decades of cryopreservation when properly adjusted for changes in laboratory testing methods over the years. These findings support evidence-based, test-at-thaw quality assessment over arbitrary expiration dates, affirming a policy approach that could expand the usable inventory of life-saving cord blood units worldwide.

## Introduction

Since Gluckman et al.^1^ reported the first successful umbilical cord blood (UCB) transplantation in a patient with Fanconi anemia in 1988, UCB has become an established source of hematopoietic stem and progenitor cells for allogeneic hematopoietic stem cell transplantation (HSCT). UCB offers several distinctive advantages over bone marrow or peripheral blood stem cell transplantation: immediate availability of cryopreserved units, tolerance of wider HLA mismatch that expands access for ethnic minorities, and a lower incidence and severity of graft-versus-host disease owing to the immunologic immaturity of UCB cells.^2^^,^^3^ Public cord blood banks worldwide now store more than 800 000 units,^4^ and more than 40 000 clinical transplantations have been performed.^5^ UCB has also been increasingly applied beyond HSCT to regenerative medicine, CAR-T cell therapy, and gene therapy,^6^^,^^7^ further enhancing the clinical value of long-term cryopreserved units. In the Republic of Korea, approximately 50 000 donor UCB units are stored across 3 government-designated public banks,^8^ including 26 903 units at the Seoul Metropolitan Government Public Cord Blood Bank as of December 2025.

Transplant success depends on infused total nucleated cell (TNC) count, CD34+ cell count, and colony-forming unit (CFU) count.^4^ Cord blood banks continuously strive to cryopreserve and supply high-quality UCB units. Post-thaw pre-transplant quality criteria, however, differ among regulatory authorities. The United States Food and Drug Administration (2014) stipulates only a TNC viability threshold (≥70%). NetCord-FACT (2024) and AABB both specify a CD34+ cell viability threshold (≥70%) and a TNC viability requirement (without a numerical threshold); a CFU assay is required by NetCord-FACT but only recommended by AABB. In Germany, pre-transplant indicators include CD45+ cell viability (≥40%), CD34+ cell viability (≥70%), TNC viability (≥60%), TNC count (≥5 × 10^8^), and CFU (≥1 colony).^9^ In Japan, the Japan Cord Blood Bank Network applies TNC recovery (≥85%), TNC viability (≥60%), CD34+ cell recovery (≥60%), CD34+ cell viability (≥80%), and CFU recovery (≥50%).^10^

Because public cord blood banks must maintain UCB units in cryogenic storage for decades, preserving quality during long-term cryopreservation is clinically important. Broxmeyer et al.^11–13^ have reported high-efficiency recovery of hematopoietic progenitor cells from UCB stored for 10, 15, and 21-23.5 years. More recently, the José Carreras Cord Blood Bank analyzed units stored up to 29 years and reported no significant deterioration with storage duration.^9^ In Korea, a single-institution study analyzed 87 UCB units stored for up to 20 years.^14^ That study used TNC recovery and TNC viability as primary indicators; post-thaw CD34+ cell viability and CFU assay—both required by AABB and NetCord-FACT—were performed only on small subsets (approximately 9 units each) as between-group comparisons of long-term cryopreserved versus fresh UCB, without establishing quantitative dose–response relationships. More recently, in a subset of the present cohort, we reported that post-thaw CD45+ and CD34+ cell viabilities remained above internationally accepted thresholds over cryopreservation periods of up to 16 years, identifying a high CD34+ cell number at cryopreservation as a key determinant of long-term unit quality.^15^ The present study extends this analysis to storage durations of up to 19 years and to hematopoietic progenitor function (total CFU), and dissociates genuine storage effects from era-related analytical variation at Korea’s largest government-designated public cord blood bank. Using CFU as the primary functional readout, we systematically evaluated the variables that influence post-thaw UCB quality in relation to storage duration, with the aim of informing pre-transplant testing and shelf-life policy for Korean public cord blood banks.

## Methods

### Study population

This retrospective cohort study included 1129 UCB units cryopreserved at the Seoul Metropolitan Government Public Cord Blood Bank that were subsequently subjected to pre-transplant testing upon transplantation request between May 2006 and December 2025. Pre-transplant testing used attached segments. Storage duration was calculated from the processing date (date of receipt) to the pre-transplant testing date, ranging from 16 days to 19.0 years (median 6.5 years, mean 6.9 years).

All UCB units were processed per the bank’s standard protocol.^16^ Units were received on average 21.9 ± 5.5 h after collection. Each unit was manually processed with pentastarch (Jeil Pharmaceutical, Seocho, Seoul, Republic of Korea) as the red blood cell sedimentation agent, followed by 2 centrifugation steps to deplete red cells and plasma. The cryoprotectant was dimethyl sulfoxide (DMSO; Cryoserve-DMSO, WAK-Chemie Medical, Steinbach, Germany) combined with 10% dextran 40 (Daehan Pharmaceutical Co., Ansan, Republic of Korea), yielding final concentrations of 10% and 1%, respectively. From November 2020, dextran 40 was replaced with Voluven (6% HES 130/0.4; Fresenius Kabi, Bad Homburg, Germany). After cryoprotectant addition, units underwent controlled-rate freezing and were stored in the liquid phase of liquid nitrogen at −196 °C.

### Post-thaw quality assessment

Thawing of the cord blood unit and segments. The thawing procedure for both the main cord blood unit and the attached segments remained unchanged throughout the entire operating period of the cord blood bank. For pre-transplant testing, the attached segment was detached from the main unit and thawed in a 37 °C water bath until the contents became liquid; owing to the small volume, thawing was completed within 10 s.

#### Complete blood count and white blood cell differential

WBC count, hemoglobin (Hb), platelet count, and WBC differential count were measured with an automated hematology analyzer (Sysmex XE-2100, Sysmex Corporation, Kobe, Japan, 2006-2020; Sysmex XN-1000, Sysmex Corporation, Kobe, Japan, from 2021).

#### CD34+ cell enumeration and viability

CD34+ cells were enumerated per the ISHAGE guidelines with a CD45-FITC/CD34-PE/7-AAD panel on the following flow cytometers: FACS Aria (BD Biosciences, San Jose, CA, USA), 2006-2010; FC-500 (Beckman Coulter, Miami, FL, USA), 2010-2022; Navios EX (Beckman Coulter, Brea, CA, USA), from 2022. Following CD34+ enumeration, viable CD45 + (G2-vCD45%, CD45^+^7-AAD^-^) and viable CD34 + (G2-vCD34%, CD34^+^7-AAD^-^) proportions were assessed by re-analyzing the acquired data with an alternative gating strategy incorporating 7-AAD viability discrimination.

#### TNC viability

TNC viability was determined by 0.4% trypan blue dye exclusion.

#### CFU assay

The CFU assay was performed with a commercial methylcellulose medium (MethoCult H4435 Enriched; StemCell Technologies, Vancouver, BC, Canada). Samples containing 1 × 10^4^ cells were suspended in 300 µL of PBS, mixed with 3 mL of methylcellulose medium, plated in duplicate on 35-mm Petri dishes, and incubated at 37 °C with 5% CO_2_ in a 100% humidified atmosphere for 14 days. Total CFU comprised all colonies irrespective of lineage (CFU-granulocyte, CFU-granulocyte/macrophage, CFU-granulocyte/erythrocyte/macrophage/megakaryocyte, and CFU-erythroid). The mean colony count of the 2 dishes was used.^17^

### Statistical analysis

The Shapiro–Wilk test was applied to all continuous variables; because most variables were non-normally distributed (Table 1), nonparametric methods were used. Recovery indicators occasionally exceeded pre-freeze values owing to flow cytometry platform transitions and increased coefficients of variation at low cell counts; both TNC recovery and CD34+ recovery were therefore truncated at 100% for analytic consistency. Correlations between storage duration and each quality indicator were assessed with Spearman rank correlation coefficients (*ρ*); linear regression slopes (*β*, units/year) were reported for effect size. Era adjustment was achieved through partial correlation by residualizing for processing date and for testing date separately.

**Table 1.** Descriptive statistics (*N* = 1129).

No.	Variable	*N*	Mean ± SD	Median (Min-Max)	Shapiro-Wilk *W*	Shapiro-Wilk *P*
	Storage					
	Storage duration (days)	1129	2508 ± 1456	2387 (16-6935)	0.9618	<.001
	Storage duration (years)	1129	6.9 ± 4.0	6.5 (0.0-19.0)	0.9618	<.001
	Pre-freeze quality					
**1**	Elapsed time (h)	1129	21.9 ± 5.5	22.0 (2.0-36.0)	0.9864	<.001
**2**	Preprocessing TNC (×10^8^)	1129	17.5 ± 5.2	16.9 (6.7-45.2)	0.9489	<.001
**3**	Postprocessing TNC (×10^8^)	1129	14.1 ± 4.2	13.6 (5.4-33.8)	0.9430	<.001
**4**	Preprocessing viability, TNC (%)	1129	98.5 ± 0.8	99.0 (90.0-99.0)	0.6086	<.001
**5**	Postprocessing viability, TNC (%)	1129	89.6 ± 2.5	90.0 (80.0-95.0)	0.9581	<.001
**6**	Preprocessing CD34+ count (×10^6^)	1129	5.6 ± 3.8	4.7 (0.2-37.7)	0.8631	<.001
**7**	Processing TNC recovery (%)	1129	80.9 ± 7.0	81.6 (58.3-96.6)	0.9810	<.001
	Post-thaw quality					
**1**	Post-thaw TNC (×10^8^)	1128	13.8 ± 4.1	13.2 (5.2-37.2)	0.9494	<.001
**2**	TNC recovery (%)	1128	95.7 ± 5.2	97.5 (64.9-100.0)	0.8183	<.001
**3**	Post-thaw viability, TNC (%)	1127	86.5 ± 2.4	86.5 (78.0-93.0)	0.9887	<.001
**4**	TNC viability recovery (%)	1127	96.5 ± 2.6	96.7 (87.0-100.0)	0.9560	<.001
**5**	CD34+ count, post-thaw (×10^6^)	1128	4.8 ± 3.2	4.1 (0.3-30.1)	0.8702	<.001
**6**	CD34+ recovery (%)	1128	82.4 ± 16.6	84.1 (11.4-100.0)	0.8985	<.001
**7**	Viable CD45 + (%)	1129	65.5 ± 13.8	66.6 (23.4-97.9)	0.9815	<.001
**8**	Viable CD34 + (%)	1129	89.9 ± 6.8	91.1 (30.0-100.0)	0.8591	<.001
**9**	Total CFU (×10^6^)	922	4.6 ± 2.7	3.9 (0.3-18.9)	0.9105	<.001
**10**	CFU per 10^6^ CD34+	922	1.0 ± 0.5	1.0 (0.2-9.5)	0.7063	<.001
**11**	CFU per 10^5^ TNC	922	329.4 ± 168.4	299.5 (35.5-1306.5)	0.9244	<.001
	Postprocessing CBC					
**1**	Hemoglobin (g/dL)	1129	17.0 ± 2.7	17.0 (8.0-26.0)	0.9774	<.001
**2**	WBC (×10³/µL)	1129	70.6 ± 20.8	67.9 (26.8-169.2)	0.9430	<.001
**3**	Platelet (×10³/µL)	1129	468.6 ± 211.9	410.0 (160.0-1990.0)	0.8326	<.001
**4**	Seg (%)	1129	60.9 ± 7.2	61.1 (35.8-87.0)	0.9978	.135
**5**	Lym (%)	1129	26.5 ± 6.8	26.1 (6.3-51.8)	0.9928	<.001
**6**	Mono (%)	1129	9.4 ± 2.2	9.2 (0.0-19.3)	0.9781	<.001
**7**	Eo (%)	1129	3.0 ± 1.7	2.6 (0.0-13.7)	0.9175	<.001
**8**	Baso (%)	1129	0.23 ± 0.22	0.20 (0.00-2.00)	0.7793	<.001
**9**	Seg (×10³/µL)	1129	43.5 ± 15.6	41.2 (10.5-131.6)	0.9192	<.001
**10**	Lym (×10³/µL)	1129	18.2 ± 5.9	17.5 (3.3-49.8)	0.9614	<.001
**11**	Mono (×10³/µL)	1129	6.6 ± 2.5	6.2 (0.0-16.7)	0.9629	<.001
**12**	Eo (×10³/µL)	1129	2.1 ± 1.3	1.8 (0.0-10.7)	0.8742	<.001
**13**	Baso (×10³/µL)	1129	0.17 ± 0.20	0.11 (0.00-1.74)	0.6947	<.001
	Post-thaw CBC					
**1**	Hemoglobin (g/dL)	1123	13.1 ± 2.1	13.0 (6.0-20.0)	0.9681	<.001
**2**	WBC (×10³/µL)	1128	55.1 ± 16.5	53.0 (3.0-148.8)	0.9519	<.001
**3**	Platelet (×10³/µL)	1124	302.7 ± 87.7	290.0 (150.0-1550.0)	0.7823	<.001
**4**	Seg (%)	1119	63.0 ± 7.7	63.1 (32.5-87.0)	0.9975	.087
**5**	Lym (%)	1118	26.0 ± 7.3	26.0 (3.3-59.6)	0.9933	<.001
**6**	Mono (%)	1127	8.0 ± 2.1	7.9 (0.0-17.6)	0.9797	<.001
**7**	Eo (%)	1127	2.9 ± 1.7	2.6 (0.2-13.5)	0.9001	<.001
**8**	Baso (%)	1071	0.10 ± 0.15	0.00 (0.00-2.20)	0.6381	<.001
**9**	Seg (×10³/µL)	1119	35.0 ± 12.7	33.0 (1.9-116.7)	0.9308	<.001
**10**	Lym (×10³/µL)	1118	13.9 ± 4.9	13.2 (0.8-46.6)	0.9495	<.001
**11**	Mono (×10³/µL)	1127	4.4 ± 1.7	4.1 (0.0-12.4)	0.9418	<.001
**12**	Eo (×10³/µL)	1127	1.6 ± 1.0	1.4 (0.1-8.4)	0.8670	<.001
**13**	Baso (×10³/µL)	1071	0.06 ± 0.10	0.00 (0.00-1.30)	0.6330	<.001

Abbreviation: TB, trypan blue.

TNC recovery, TNC viability recovery, and CD34+ recovery were capped at 100% (see Methods); occasional post-thaw values exceeding the pre-freeze value due to flow cytometry platform transition or low-count coefficient-of-variation increases were truncated at 100% for analytic consistency.

To see which factors best explain a cord blood unit’s colony-forming capacity, we used a machine-learning model (Random Forest) that can capture complex, nonlinear relationships. Predictors of CFU were assessed in a multivariate framework with Random Forest regression (500 trees, 19 features; 10-fold cross-validation *R*^2^ = 0.702 ± 0.155). Feature importance was reported by Mean Decrease Impurity and by permutation importance. Paired pre- vs post-thaw comparisons used the paired *t*-test. Analyses were performed in Python 3.10 (scipy, sklearn); statistical significance was *P* < .05, with Bonferroni correction where multiple comparisons were performed.

## Results

### Descriptive statistics

Descriptive statistics for the 1129 UCB units are presented in Table 1. Storage duration averaged 2508 ± 1456 days (median 2387 days; 6.9 ± 4.0 years; range 0.0-19.0 years). Shapiro–Wilk tests confirmed non-normal distributions (*P* < .001) for all variables except postprocessing Seg% (*P* = .135) and post-thaw Seg% (*P* = .087). Pre-freeze quality parameters (7 indicators; elapsed time, TNC counts preprocessing and postprocessing, TNC preprocessing and postprocessing viabilities, CD34+ count, and processing TNC recovery) and post-thaw quality parameters (eleven indicators, including post-thaw TNC, TNC recovery, TNC viability, CD34+ count, CD34+ recovery, viable CD45+ and CD34+, total CFU [*N* = 922], CFU per 10^6^ CD34+ cells, and CFU per 10^5^ TNC) are detailed in Table 1. Paired pre- vs post-thaw complete blood count (CBC) changes were analyzed for thirteen indicators.

### Correlations between quality indicators and storage duration

#### Pre-freeze quality

In unadjusted analyses, elapsed time showed a weak positive correlation with storage duration (*ρ* = +0.132, *β* = +0.20 h/year, *P* < .001), which persisted after testing-date adjustment (*ρ* = +0.376, *P* < .001; Table 2). Preprocessing CD34+ count showed a negative correlation (*ρ* = −0.107, *P* < .001) that strengthened after testing-date adjustment (*ρ* = −0.216, *P* < .001). Postprocessing TNC viability (trypan blue) and processing TNC recovery showed significant negative correlations in the raw analysis (*ρ* = −0.094, *P* = .002; *ρ* = −0.177, *P* < .001); these were abolished after processing-date adjustment and reappeared with greater magnitude after testing-date adjustment (*ρ* = −0.262 and −0.440, both *P* < .001).

**Table 2:** Spearman correlation with storage duration.

		Unadjusted			Processing-date adjusted			Testing-date adjusted		
Variable	*N*	*ρ*	%/year	*P*	*ρ*	%/year	*P*	*ρ*	%/year	*P*
**Pre-freeze quality**										
** Elapsed time (h)**	1129	+0.132	+0.90	<.001	−0.014	−0.09	.639	+0.376	+2.57	<.001
** Preprocessing TNC (×10^8^)**	1129	+0.037	+0.27	.212	+0.029	+0.16	.334	+0.045	+0.45	.129
** Preprocessing viability, TB (%)**	1129	+0.001	−0.001	.970	+0.019	−0.002	.529	+0.033	+0.001	.261
** Preprocessing CD34+ count (×10^6^)**	1129	−0.108	−1.98	<.001	−0.023	−0.89	.439	−0.215	−3.82	<.001
** Postprocessing TNC (×10^8^)**	1129	−0.018	−0.18	.551	+0.031	+0.17	.295	−0.089	−0.77	.003
** Postprocessing viability, TNC (%)**	1129	−0.094	−0.08	.002	+0.006	−0.001	.845	−0.262	−0.20	<.001
** Processing TNC recovery (%)**	1129	−0.177	−0.43	<.001	+0.015	+0.01	.606	−0.440	−1.16	<.001
**Post-thaw quality**										
** Post-thaw TNC (×10^8^)**	1128	+0.037	+0.24	.215	+0.059	+0.43	.049	−0.006	−0.06	.851
** TNC recovery (%)**	1128	+0.195	+0.26	<.001	+0.115	+0.19	<.001	+0.233	+0.38	<.001
** Post-thaw viability, TB (%)**	1127	−0.170	−0.12	<.001	−0.096	−0.06	.001	−0.250	−0.21	<.001
** TNC viability recovery (%)**	1127	−0.056	−0.03	.061	−0.070	−0.04	.018	−0.000	−0.005	.991
** CD34+ count, post-thaw (×10^6^)**	1128	+0.016	+0.003	.587	+0.060	+0.77	.044	−0.045	−1.28	.131
** CD34+ recovery (%)**	1128	+0.194	+1.14	<.001	+0.136	+1.01	<.001	+0.277	+1.37	<.001
** Viable CD45 + (%)**	1129	+0.342	+1.71	<.001	+0.529	+2.75	<.001	+0.014	−0.04	.640
** Viable CD34 + (%)**	1129	−0.151	−0.17	<.001	−0.206	−0.25	<.001	+0.047	−0.02	.116
** Total CFU (×10^6^)**	922	−0.160	−2.34	<.001	−0.171	−2.65	<.001	−0.082	−1.68	.013
** CFU per 10^5^ TNC**	922	−0.197	−2.61	<.001	−0.224	−3.17	<.001	−0.086	−1.40	.009
** CFU per 10^6^ CD34+**	922	−0.238	−2.97	<.001	−0.333	−4.20	<.001	−0.032	−0.33	.327
**Paired delta (%) and decrease rate (%) of WBC differential types** ^a^										
** ΔHb (g/dL)**	1123	−0.140	−0.04	<.001	−0.246	−0.08	<.001	+0.077	0.02	.010
** ΔWBC (×10³/µL)**	1128	+0.143	+0.27	<.001	+0.048	+0.12	.108	+0.259	+0.51	<.001
** ΔPlt (×10³/µL)**	1124	−0.127	−7.52	− <.001	−0.008	−1.08	.799	−0.309	−18.35	<.001
** ΔSeg (%p)**	1119	+0.391	+0.33	<.001	+0.519	+0.47	<.001	+0.057	+0.09	.056
** ΔLym (%p)**	1118	−0.333	−0.32	<.001	−0.476	−0.47	<.001	−0.014	−0.05	.629
** ΔMono (%p)**	1127	−0.036	−0.01	.226	−0.014	+0.00	.636	−0.040	−0.02	.180
** ΔEo (%p)**	1127	+0.035	+0.01	.242	+0.057	+0.01	.054	+0.015	+0.01	.608
** ΔBaso (%p)**	1071	−0.161	−0.01	<.001	−0.131	−0.01	<.001	−0.102	−0.01	<.001
** Seg change rate (%)**	1119	+0.400	0.78	<.001	+0.402	0.82	<.001	+0.282	0.69	<.001
** Lym change rate (%)**	1118	−0.184	−0.65	<.001	−0.346	−1.28	<.001	+0.123	0.42	<.001
** Mono change rate (%)**	1124	+0.034	0.46	.256	+0.028	+0.37	.356	+0.056	0.62	.059
** Eo change rate (%)**	1114	+0.096	0.39	.001	+0.104	0.33	<.001	+0.099	0.49	<.001
** Baso change rate (%)**	883	−0.159	−1.90	<.001	−0.232	−2.87	<.001	+0.037	0.49	.267

Δ = post-thaw value − postprocessing value. For differential components, Δ is in percentage points (%p); for direct measurements, in native units (ΔHb, g/dL; ΔWBC and ΔPlt, ×10³/µL). Decrease rate (%) = (post-thaw absolute count − postprocessing absolute count)/post-thaw absolute count × 100; absolute count = WBC × differential %/100 (×10³/µL). Positive values indicate a decrease after thawing. *ρ* = Spearman rank correlation with storage duration; processing-/testing-date adjusted values are partial Spearman correlations controlling for the respective calendar variable.

a For paired delta and change rate variables, the %/year columns report the regression slope *β* (per-year change) in native units (%p/year for Δ differential percentages; %/year for decrease rates; g/dL/year or ×10³/µL/year for ΔHb, ΔWBC, ΔPlt), rounded to 2 decimals.

#### Post-thaw quality

In unadjusted analyses, TNC recovery (*ρ* = +0.194, *P* < .001), CD34+ recovery (*ρ* = +0.194, *P* < .001), and viable CD45 + (*ρ* = +0.342, *P* < .001) showed significant positive correlations with storage duration. Conversely, post-thaw TNC viability (trypan blue; *ρ* = −0.170) and viable CD34 + (*ρ* = −0.151) showed negative correlations that became more pronounced after testing-date adjustment (*ρ* = −0.250 and +0.047, respectively).

**Figure 1.**
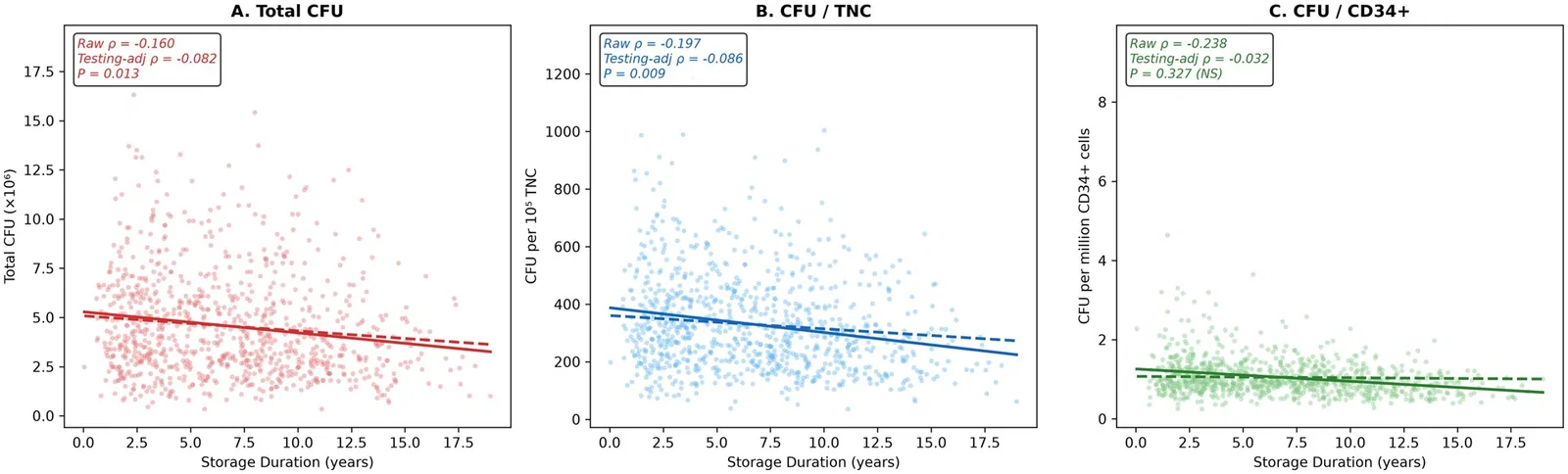
Storage duration and CFU quality metrics with era-adjusted analysis (*N* = 922). Scatter plots of (A) total CFU (×10^6^), (B) CFU per 10^5^ TNC, and (C) CFU per million CD34+ cells against storage duration (0-19 years), with linear regression lines (solid, raw [unadjusted]; dashed, testing-date adjusted). Each dot represents 1 cord blood unit. Inset boxes show Spearman *ρ* for raw (unadjusted) and testing-date adjusted analyses, with the 2-sided *P* value of the adjusted correlation. All 3 metrics were significantly negatively correlated with storage before adjustment (raw *ρ*: −0.160 to −0.238; all *P* < .001). After testing date adjustment, total CFU (*P* = .013) and CFU/TNC (*P* = .009) retained attenuated but significant correlations, whereas CFU/CD34+ became nonsignificant (*ρ* = −0.032, *P* = .327). Complete raw, processing-date, and testing-date–adjusted correlations and regression slopes (*β*) are provided in Table 2. CFU, colony-forming unit; TNC, total nucleated cell; NS, not significant.

Total CFU (*ρ* = −0.160, *β* = −0.11 × 10^6^/year, *P* < .001), CFU per 10^5^ TNC (*ρ* = −0.197, *β* = −8.59/year, *P* < .001), and CFU per 10^6^ CD34 + (*ρ* = −0.238, *β* = −0.0311/year, *P* < .001) all showed significant negative correlations with storage duration before adjustment. These correlations strengthened after processing-date adjustment (*ρ* = −0.171, −0.224, and −0.333; all *P* < .001). After testing-date adjustment, total CFU and CFU per 10^5^ TNC were substantially attenuated (*ρ* = −0.082, *P* = .013; *ρ* = −0.086, *P* = .009), whereas the negative correlation between CFU per 10^6^ CD34+ and storage duration was abolished (*ρ* = −0.032, *P* = .327; Figure 1).

#### Paired delta and decrease rate of WBC differential types

In unadjusted analyses, ΔWBC showed a weak positive correlation with storage duration (*ρ* = +0.143, *β* = +0.27 × 10³/µL per year, *P* < .001), whereas ΔHb (*ρ* = −0.140, *β* = −0.04 g/dL per year, *P* < .001) and ΔPlatelet (*ρ* = −0.127, *β* = −7.52 × 10³/µL per year, *P* < .001) showed negative correlations. Among differential components, ΔSeg (*ρ* = +0.391, *β* = +0.33%p/year, *P* < .001) and ΔLym (*ρ* = −0.333, *β* = −0.32%p/year, *P* < .001) showed associations that strengthened after processing-date adjustment (*ρ* = +0.519, *β* = +0.47%p/year and *ρ* = −0.476, *β* = −0.47%p/year, respectively). ΔBaso showed a significant negative correlation (*ρ* = −0.161, *β* = −0.01%p/year, *P* < .001), whereas ΔMono (*P* = .226) and ΔEo (*P* = .242) did not reach significance. The Seg decrease rate (*ρ* = +0.4, *β* = +0.78%/year, *P* < .001) had the positive correlation, whereas Baso (*ρ* = −0.159, *β* = −1.90%/year, *P* < .001) and Lym (*ρ* = −0.184, *β* = −0.65%/year, *P* < .001) decrease rates had negative correlations. All direct CBC measurements decreased significantly from postprocessing (pre-freeze) to post-thaw (all paired *P* < .001).

### Univariate and multivariate analysis of CFU predictors

**Figure 2.**
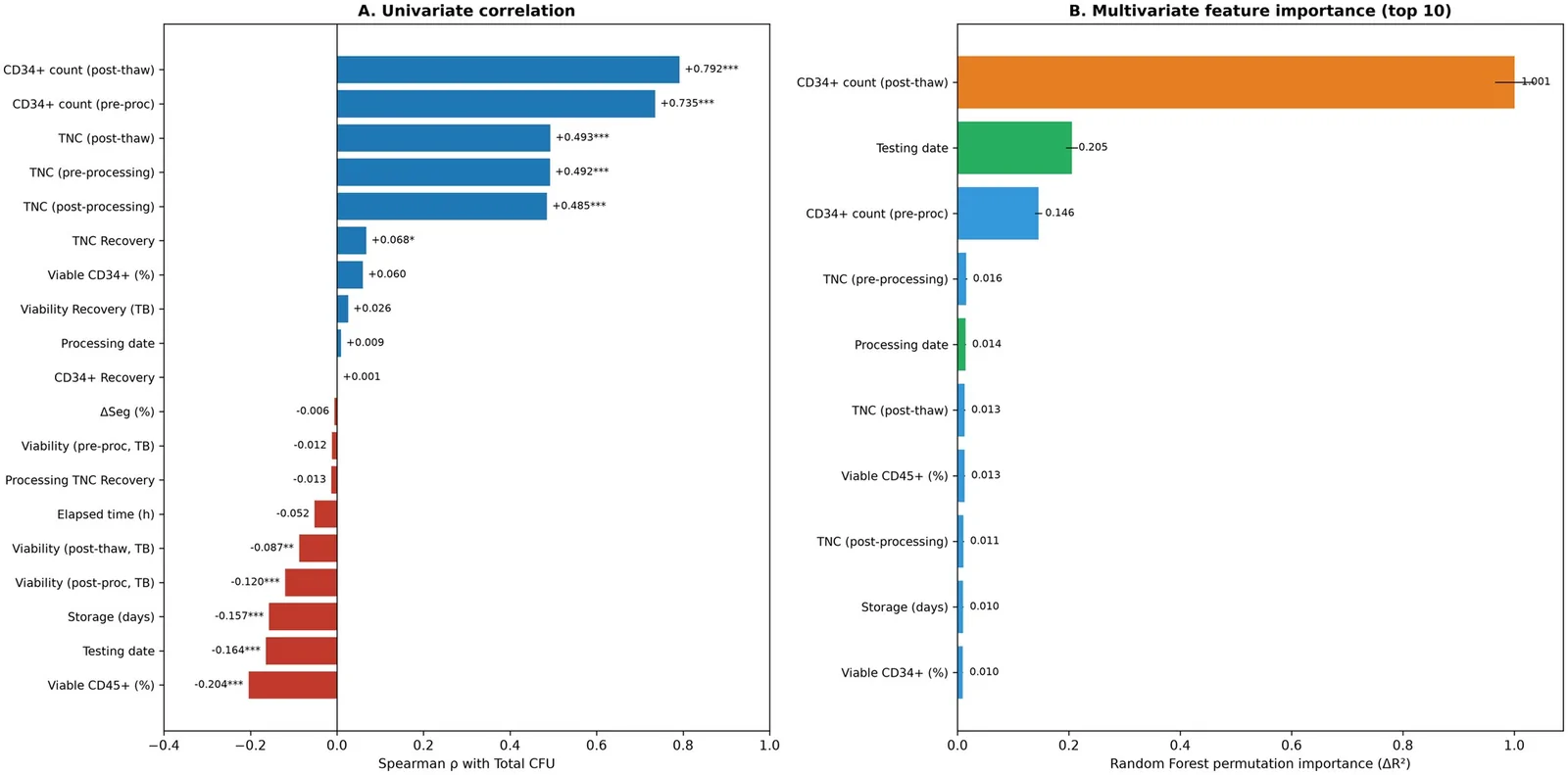
CFU explanatory variables: univariate correlation vs multivariate feature importance. (A) Horizontal bar chart of univariate Spearman rank correlation coefficients between each quality variable and total CFU (*N* = 915). Bars extending to the right indicate positive correlations and bars extending to the left indicate negative correlations (shown in blue and red, respectively). (B) Top 10 variables (multivariate feature importance) ranked by Random Forest permutation importance (Δ*R*^2^) from the RF regression model (19 features; *N* = 915; 10-fold cross-validation *R*^2^ = 0.702 ± 0.155). Error bars, SD from 10 permutation rounds. Testing date ranks No. 2 by RF but only No. 7 by univariate Spearman |*ρ*| (bars in (A) are ordered by signed *ρ*), reflecting its nonlinear/era-dependent contribution.

In unadjusted univariate analyses, the strongest positive correlate with total CFU was post-thaw CD34+ count (*ρ* = +0.793, *P* < .001), followed by preprocessing CD34+ count (*ρ* = +0.737; Figure 2 and Tables S1 and S2). TNC-related variables showed moderate positive correlations (*ρ* ≈ +0.49). Viable CD45 + (*ρ* = −0.211) and postprocessing TNC viability (*ρ* = −0.119) showed significant negative correlations, and storage duration showed a weak negative correlation (*ρ* = −0.160; all *P* < .001). Among absolute-count differences of WBC components, ΔMono abs (*ρ* = −0.295) and ΔSeg abs (*ρ* = −0.230) were significantly negatively correlated with CFU, whereas proportional changes (ΔSeg%, ΔLym%, ΔEo%) were not.

After processing-date adjustment, the strengths of CD34+- and TNC-related predictors were essentially unchanged (post-thaw CD34+ *ρ* = +0.796; preprocessing CD34+ *ρ* = +0.763; post-thaw TNC *ρ* = +0.497; all *P* < .001). After testing date adjustment, post-thaw CD34 + (*ρ* = +0.823) and preprocessing CD34 + (*ρ* = +0.763) became even stronger, whereas storage duration (*ρ* = −0.160 → −0.082, *P* = .013) and viable CD45 + (*ρ* = −0.211 → −0.134, *P* < .001) were attenuated, indicating era-effect mediation.

The Random Forest model explained about 70% of the variation in CFU activity (10-fold cross-validation *R^2^* = 0.702 ± 0.155). The top 5 predictors were post-thaw CD34+ count (Δ*R*^2^ = 1.001), testing date (Δ*R*^2^ = 0.205), preprocessing CD34+ count (Δ*R*^2^ = 0.146), preprocessing TNC (Δ*R*^2^ = 0.016), and processing date (Δ*R*^2^ = 0.014). Notably, the importance of testing date rose from seventh place in univariate Spearman rank (*ρ* = −0.164) to second place in Random Forest analysis, and processing date rose from 17th place (*ρ* = +0.009) to fifth place. This indicates that these variables contribute through nonlinear and interactive pathways.

## Discussion

This is the largest study to date—1129 UCB units cryopreserved for up to 19 years at a single institution using manual processing—to evaluate post-thaw quality with total CFU as the primary outcome. Beyond confirming preservation, the novel contribution here is the era (processing-date and testing-date) adjustment that isolates the intrinsic effect of storage, together with the CFU/CD34 + (and post-thaw CD34+) potency framework.

The 3 principal findings are: (1) per-CD34+ cell colony-forming capacity (CFU/CD34+) is preserved independently of storage duration after testing-date adjustment (*P* = .327), confirming that unit-level hematopoietic progenitor cell function is retained up to 19 years of cryopreservation; (2) post-thaw CD34+ count is the strongest CFU predictor (*ρ* = +0.793; ranked first in Random Forest analysis), and this relationship is robust to processing- and testing-date adjustment; and (3) the isolated effect of storage duration is modest (unadjusted *ρ* = −0.160 → testing-date-adjusted ρ = −0.082), with much of the apparent storage-related change in unadjusted metrics instead reflecting era effects associated with processing and testing dates.

Because CFU measurement is difficult to standardize and requires a 14-day culture, post-thaw CD34+ count is the routinely used quality indicator, and international standards treat CFU as a growth/no-growth determination. Our data show that post-thaw CD34+ count is the single variable best explaining post-thaw CFU, supporting its role as a reliable surrogate when CFU is preserved.^18^ Preprocessing CD34+ count also correlated strongly with post-thaw CFU (*ρ* = +0.737), supporting its utility as a pre-freeze predictor at the banking stage. TNC-related indicators showed only moderate predictive strength (*ρ*  ≈  0.49), indicating that CD34+ provides independent and superior information. That CD34+ count is the strongest single predictor of total CFU reflects a quantitative relationship; CFU/CD34+ normalization provides complementary qualitative information on per-cell colony-forming capacity, which was essentially preserved over 19 years after era adjustment (*β*  ≈  0; *P* = .327).

After testing-date adjustment, CFU/CD34+ lost its association with storage duration (*β* = −0.33%/year, *P* = .327), whereas CFU/TNC retained a small residual negative correlation (*β* = −1.40%/year, *P* = .009). This residual is minor (explaining <1% of the variance) and is largely attributable to testing-date era effects; accordingly, we regard CFU/CD34+, which directly reflects per-cell progenitor function, as the more appropriate metric of long-term potency.

An important methodological finding is that both processing date and testing date must be treated as independent confounders when analyzing storage-duration effects. Although storage duration equals testing date minus processing date, these 2 calendar variables act through independent era-associated pathways. Processing-date confounders include regulatory policy changes and changes in banking reception and processing practices (eg, manual vs automated processing; replacement of red cell sedimentation agents and cryoprotectants). Testing-date confounders include changes in equipment and reagents (eg, dilution ratios for attached segments, automated hematology analyzers, flow cytometer platform transitions, and CFU culture conditions). In the Random Forest analysis, testing date and processing date emerged as far more influential than their simple correlations had suggested: testing date rose from seventh to second in importance and processing date from 17th to 5th (Figure 2B and Figure S2 [see online supplementary material for a color version of this figure] and Table S2). In other words, the calendar era in which a unit was processed and tested affects measured CFU through nonlinear and interacting pathways that pairwise correlations cannot capture. Practically, this means that reporting simple Spearman correlations without accounting for processing and testing dates can overstate apparent quality deterioration with long-term storage. Partial-correlation or multivariate approaches should therefore become the standard analytical framework when evaluating cord blood unit quality over time.

Our findings are directionally consistent with prior literature. Broxmeyer et al.^11–13^^,^^19^ reported preserved CFU recovery in UCB after 10, 15, 21-23.5, and 27 years of cryopreservation, and Hussein et al.^20^ reported that CFU-GM per million NC was not significantly related to storage duration. The present study extends these reports by mechanistically explaining the dissociation between CFU/TNC and CFU/CD34+ in a large long-term cohort (*N* = 1129, up to 19 years). In a clinical framework, NetCord-FACT CD34+ cell viability (≥70%) compliance was 98.4%, and composite compliance with the German criteria was 96.2% (887/922), exceeding 93% in all 4 storage-duration bins (Table S3). Composite compliance with the Japanese criteria was 56.5%, driven largely by CD45+ cell viability, which is difficult to standardize across laboratories.^21^^,^^22^ In clinical release practice, units with marginal individual indicators can still be released following consultation with the transplant center using per-recipient body-weight-normalized TNC and CD34+ indicators. Because Korea has no unified pre-transplant quality standard, a minimum national standard should be established.

Neutrophils are reported to be particularly vulnerable to freeze-thaw injury, with marked loss of function. In our cohort the automated post-thaw segmented-neutrophil fraction appeared to rise; we interpret this as a discordance between morphology and function rather than true neutrophil survival, because an automated differential enumerates cells morphologically and does not capture viability. Apoptosis-based phenotyping (eg, Annexin V/7-AAD) is warranted in future work to resolve this morphology—function discordance.

Below the glass transition temperature of water (∼−130 °C), molecular-level biological activity ceases, and cryopreserved cells can theoretically be stored indefinitely.^23^^,^^24^ Scaradavou recently stated that cord blood stem cells “do not age” under appropriate banking conditions.^25^ Korea currently has no defined shelf-life (expiration) limit for banked cord blood units. Our findings offer an evidence base for any such policy that may be established in the future. Once differences in testing era are accounted for, a unit’s colony-forming capacity per CD34+ cell remained essentially unchanged even after nearly 2 decades in storage (*β* = −0.33%/year, *P* = .327). Consistent with this, storage duration was a minor contributor to CFU activity—ranking only ninth of 19 variables in the Random Forest model (Δ*R*^2^ = 0.0097) and falling well behind the factors that truly matter, namely the post-thaw CD34+ count, the testing era, and the preprocessing CD34+ count. In practical terms, not how long it has been frozen but how many viable stem cells a unit is what determines its expected potency. These data suggest that, should a shelf-life limit be considered, storage duration alone provides little justification for discarding otherwise high-quality units. Furthermore, given the limited sample size in the 15-19-year bin (*n* = 36), a shift from a “duration-based” to a “test-based” framework is reasonable: suitability for release should be judged unit-by-unit at the time of transplantation request using composite German/FACT-NetCord criteria (viable CD45+ ≥40%, viable CD34+ ≥70%, TNC viability, post-thaw TNC count, and CFU growth). Pre-transplant testing policy and shelf-life policy are mutually reinforcing: as pre-transplant testing is systematically implemented, a conservative duration cap becomes less necessary; as shelf-life extends, pre-transplant testing rigor becomes more important.

Limitations include: (1) direct linkage to post-transplant outcomes (engraftment rate, time to neutrophil recovery) was not possible, although CFU is an established surrogate; (2) pre-transplant testing used the attached integral segment rather than the main bag. Although the contiguous segment is widely used as a surrogate for the unit and segment and bag cell counts are strongly correlated, several groups have reported that the absolute values of the segment may contain higher post-thaw CD34+ cells and CFU-GM than the bag.^26^^,^^27^ The present results should therefore be interpreted with due caution regarding absolute values, and prospective paired segment-bag validation remains warranted; (3) adjustment for processing and testing dates across a 19-year span is partial and cannot fully eliminate era effects—a longitudinal design analyzing the same specimens over time would be more precise,^28^ and this is a topic for future research.

## Conclusions

From a retrospective analysis of 1129 UCB units cryopreserved for up to 19 years at Korea’s largest government-designated public cord blood bank, 3 conclusions emerge. First, after testing-date era adjustment, CFU/CD34+ is preserved independent of storage duration, confirming that per-CD34+ cell colony-forming capacity is substantially preserved during long-term cryopreservation; post-thaw CD34+ count is the single strongest CFU and a reliable clinical surrogate that can substitute for the 14-day CFU assay. Second, much of the apparent correlation between storage duration and quality indicators reflects era effects from processing and testing dates, and future long-term stability studies should adopt partial correlation or multivariate era-adjustment as standard methodology. Third, composite compliance with German/FACT-NetCord pre-transplant quality criteria was maintained above 93% in every storage-duration bin (overall 96.2%), and storage duration itself was a secondary predictor of CFU, providing scientific grounds for shifting from a duration-based to a test-based shelf-life policy. Because Korea has not yet defined a shelf-life limit or standardized pre-transplant quality criteria, establishing the two as mutually reinforcing, evidence-based standards is essential; their coordinated implementation could increase clinical utilization of Korea’s cord blood resources while minimizing unnecessary disposal.

## Supplementary Material

szag075_Supplementary_Data

## Data Availability

The de-identified data supporting the findings of this study are available from the corresponding author upon reasonable request.
