# Supplementary material for: Up to 19-year cryopreservation does not impair hematopoietic progenitor function in cord blood: a 1129-unit analysis from the largest Korean public cord blood bank
Source: Stem Cells Transl Med. 2026 Aug 25;15(9):szag075. doi: 10.1093/stcltm/szag075 (PMC13505624; doi:10.1093/stcltm/szag075)
Supplement: Supplementary file 1 [file szag075_supplementary_data.pdf]

Supplemental Information

Up to 19-Year Cryopreservation Does Not Impair Hematopoietic Progenitor Function in Cord Blood: 1,129-Unit Analysis from the Largest Korean Public CBB

Eun Youn Roh[a,b,c,1], Hye Ryun Lee[d,1], Sue Shin[a,b,c,*], Jong Hyun Yoon[a,b,c], Nam-Hee Kim[a,c], Hyun-Woong Park[a,c], Byoung Jae Kim[b,e]

[a]Department of Laboratory Medicine, SMG-SNU Boramae Medical Center, Seoul, Republic of Korea

[b]Seoul Metropolitan Government Public Cord Blood Bank (ALLCORD), Seoul, Republic of Korea

[c]Department of Laboratory Medicine, Seoul National University College of Medicine, Seoul, Republic of Korea

[d]Department of Laboratory Medicine, National Medical Center, Seoul, Republic of Korea

[e]Department of Obstetrics and Gynecology, SMG-SNU Boramae Medical Center, Seoul, Republic of Korea

[1]These authors contributed equally to this study.

***Corresponding author:** Sue Shin, MD, PhD
Department of Laboratory Medicine, Seoul National University Boramae Medical Center
20 Boramaero 5-gil, Dongjak-gu, Seoul 07061, Republic of Korea
E-mail: jeannie@snu.ac.kr

Table of Contents

Supplemental Table S1. Spearman Correlation of Each Variable with Total CFU

Supplemental Table S2. Random Forest Regression: Feature Importance for CFU Prediction

Supplemental Table S3. Compliance with Pre-Transplant Quality Criteria, Stratified by Storage Duration (N = 1,129)

Supplemental Figure S1. Spearman correlation of quality variables with Total CFU: raw vs. era-adjusted (N = 922)

Supplemental Figure S2. Identifying the factors that predict colony-forming capacity (CFU) using Random Forest regression (N = 915)

Supplemental Table S1. Spearman Correlation of Each Variable with Total CFU

Variable	N	Unadjusted		Processing-date adjusted		Testing-date adjusted	
		ρ	P	ρ	P	ρ	P
Pre-freeze							
Elapsed time	922	-0.0564	0.0871	-0.0530	0.1080	-0.0820	0.0127
TNC (pre-proc)	922	+0.4955	<0.0001	+0.4969	<0.0001	+0.5024	<0.0001
Viability (pre-proc, TB)	922	-0.0064	0.8472	-0.0003	0.9919	-0.0009	0.9785
CD34+ count (pre-proc)	922	+0.7367	<0.0001	+0.7634	<0.0001	+0.7629	<0.0001
TNC (post-proc)	922	+0.4887	<0.0001	+0.4921	<0.0001	+0.5098	<0.0001
Viability (post-proc, TB)	922	-0.1191	0.0003	-0.1320	<0.0001	-0.1014	0.0020
Processing TNC recovery	922	-0.0083	0.8020	-0.0318	0.3349	+0.0220	0.5044
Post-thaw							
TNC (post-thaw)	922	+0.4960	<0.0001	+0.4965	<0.0001	+0.5139	<0.0001
TNC recovery	922	+0.0653	0.0475	+0.0650	0.0486	+0.0802	0.0148
CD34+ count (post-thaw)	922	+0.7926	<0.0001	+0.7956	<0.0001	+0.8230	<0.0001
CD34+ recovery	922	-0.0005	0.9877	+0.0060	0.8566	+0.0091	0.7826
Viable CD45+ (%)	922	-0.2113	<0.0001	-0.2273	<0.0001	-0.1340	<0.0001
Viable CD34+ (%)	922	+0.0536	0.1036	+0.0556	0.0915	+0.0228	0.4891
TNC Viability (post-thaw)	921	-0.0898	0.0064	-0.0995	0.0025	-0.0823	0.0125
TNC Viability recovery	921	+0.0242	0.4637	+0.0274	0.4065	+0.0128	0.6970
Paired Delta							
ΔLym%	915	+0.0637	0.0541	+0.0693	0.0361	-0.0108	0.7444
ΔMono%	921	-0.0949	0.0040	-0.0949	0.0040	-0.0957	0.0036
ΔEo%	921	-0.0297	0.3678	-0.0296	0.3692	-0.0125	0.7059
ΔBaso%	867	-0.0766	0.0241	-0.0861	0.0112	-0.1067	0.0017
ΔLym abs	915	-0.0446	0.1779	-0.0537	0.1045	-0.1192	0.0003
ΔMono abs	921	-0.2945	<0.0001	-0.2937	<0.0001	-0.3029	<0.0001
ΔSeg abs	916	-0.2298	<0.0001	-0.2291	<0.0001	-0.2115	<0.0001
ΔHb	917	+0.0361	0.2742	+0.0435	0.1880	-0.0226	0.4940
Storage							
Storage duration (yr)	922	-0.1596	<0.0001	-0.1709	<0.0001	-0.0820	0.0127

Note. ρ = Spearman rank correlation with Total CFU. Era adjustment by partial correlation, residualising for processing date and for testing date separately. Recovery indicators truncated at 100%. Denominators vary with the availability of each measurement.

Supplemental Table S2. Random Forest Regression: Feature Importance for CFU Prediction

Rank	Feature	Spearman ρ	Spearman P	Spearman Rank	MDI Importance	MDI Rank	Permutation Importance ± SD	Perm Rank
1	CD34+ count (post-thaw)	+0.7916	<0.0001	1	0.6218	1	1.0007 ± 0.0354	1
2	Testing date	-0.1645	<0.0001	7	0.1080	2	0.2054 ± 0.0107	2
3	CD34+ count (pre-proc)	+0.7354	<0.0001	2	0.1066	3	0.1455 ± 0.0064	3
4	TNC (pre-processing)	+0.4922	<0.0001	4	0.0191	4	0.0158 ± 0.0012	4
5	Processing date	+0.0093	0.7776	17	0.0147	5	0.0144 ± 0.0007	5
6	TNC (post-thaw)	+0.4929	<0.0001	3	0.0140	6	0.0128 ± 0.0010	6
7	Viable CD45+ (%)	-0.2042	<0.0001	6	0.0139	7	0.0128 ± 0.0005	7
8	TNC (post-processing)	+0.4855	<0.0001	5	0.0120	9	0.0107 ± 0.0007	8
9	Storage (days)	-0.1568	<0.0001	8	0.0104	11	0.0102 ± 0.0005	9
10	Viable CD34+ (%)	+0.0600	0.0697	12	0.0118	10	0.0097 ± 0.0004	10
11	Processing TNC Recovery	-0.0129	0.6978	15	0.0120	8	0.0096 ± 0.0006	11
12	TNC Recovery	+0.0676	0.0410	11	0.0084	14	0.0094 ± 0.0007	12
13	ΔSeg (%)	-0.0057	0.8635	18	0.0090	13	0.0078 ± 0.0004	13
14	Viability Recovery (TB)	+0.0257	0.4380	14	0.0095	12	0.0077 ± 0.0004	14
15	CD34+ Recovery	+0.0013	0.9681	19	0.0077	15	0.0073 ± 0.0004	15
16	Elapsed time (h)	-0.0516	0.1188	13	0.0068	16	0.0055 ± 0.0003	16
17	Viability (post-thaw, TB)	-0.0874	0.0081	10	0.0068	17	0.0054 ± 0.0003	17
18	Viability (post-proc, TB)	-0.1199	0.0003	9	0.0051	18	0.0036 ± 0.0002	18
19	Viability (pre-proc, TB)	-0.0116	0.7255	16	0.0024	19	0.0018 ± 0.0002	19

Note. Random Forest regression (500 trees, 19 features; N = 915; 10-fold cross-validation $R^2 = 0.702 \pm 0.155$). MDI = mean decrease in impurity. Permutation importance from 30 rounds. Spearman Rank is by |ρ|. Spearman ρ = univariate Spearman rank correlation; Random Forest captures multivariate, non-linear relationships beyond pairwise correlations.
Key finding: Testing date ranks #2 by RF permutation importance but only #7 by Spearman |ρ|, reflecting its non-linear confounding effect on CFU. Processing date ranks #5 by RF but #17 by Spearman |ρ|, further supporting era-dependent protocol changes as a confounder.

Supplemental Table S3. Compliance with Pre-Transplant Quality Criteria, Stratified by Storage Duration (N = 1,129)

Criterion	Cutoff	Overall	0–5 yr (n=438)	5–10 yr (n=435)	10–15 yr (n=220)	15–19 yr (n=36)
German Criteria (Liedtke et al., 2024)						
CD45+ cell viability	≥40%	1095/1129 (97.0%)	416/438 (95.0%)	424/435 (97.5%)	219/220 (99.5%)	36/36 (100.0%)
CD34+ cell viability	≥70%	1111/1129 (98.4%)	431/438 (98.4%)	430/435 (98.9%)	216/220 (98.2%)	34/36 (94.4%)
TNC viability (Trypan blue)	≥60%	1127/1127 (100.0%)	437/437 (100.0%)	435/435 (100.0%)	219/219 (100.0%)	36/36 (100.0%)
Post-thaw TNC	≥5×10^8	1128/1128 (100.0%)	438/438 (100.0%)	435/435 (100.0%)	219/219 (100.0%)	36/36 (100.0%)
CFU	≥1 colony	922/922 (100.0%)	376/376 (100.0%)	332/332 (100.0%)	185/185 (100.0%)	29/29 (100.0%)
All German criteria met	—	887/922 (96.2%)	354/376 (94.1%)	326/332 (98.2%)	180/185 (97.3%)	27/29 (93.1%)
Japanese Criteria (JCBBN)						
TNC viability (7-AAD, =vCD45+)	≥60%	743/1129 (65.8%)	223/438 (50.9%)	292/435 (67.1%)	196/220 (89.1%)	32/36 (88.9%)
CD34+ cell viability	≥80%	1055/1129 (93.4%)	413/438 (94.3%)	406/435 (93.3%)	202/220 (91.8%)	34/36 (94.4%)
TNC recovery	≥85%	1074/1128 (95.2%)	411/438 (93.8%)	416/435 (95.6%)	211/219 (96.3%)	36/36 (100.0%)
CFU recovery	≥50%	N/A‡	N/A‡	N/A‡	N/A‡	N/A‡
CD34+ cell recovery	≥60%	1017/1128 (90.2%)	370/438 (84.5%)	400/435 (92.0%)	211/219 (96.3%)	36/36 (100.0%)
All Japanese criteria met§	—	637/1128 (56.5%)	191/438 (43.6%)	243/435 (55.9%)	172/219 (78.5%)	31/36 (86.1%)

* German TNC viability criterion uses Fluoroquench under fluorescent microscopy (Liedtke et al., 2024); our method used Trypan blue exclusion as the comparable dye-exclusion assay.
† Japanese TNC viability criterion (JCBBN) is measured by flow cytometry with 7-AAD staining on CD45+ cells; corresponds to our viable CD45+ (%) measurement.
‡ CFU recovery not available: pre-freeze CFU assay was not routinely performed.
§ "All Japanese criteria met" excludes CFU recovery from the composite because pre-freeze CFU was not available.
Denominators vary across criteria due to differential availability of individual measurements (e.g., CFU assay available in 922/1,129 units).
Cell frequency differences between bins reflect both the era of banking activity and the timing of pre-transplant testing requests.
Note. Storage duration was calculated from the date of cord blood processing (receipt) to the date of pre-transplant testing.

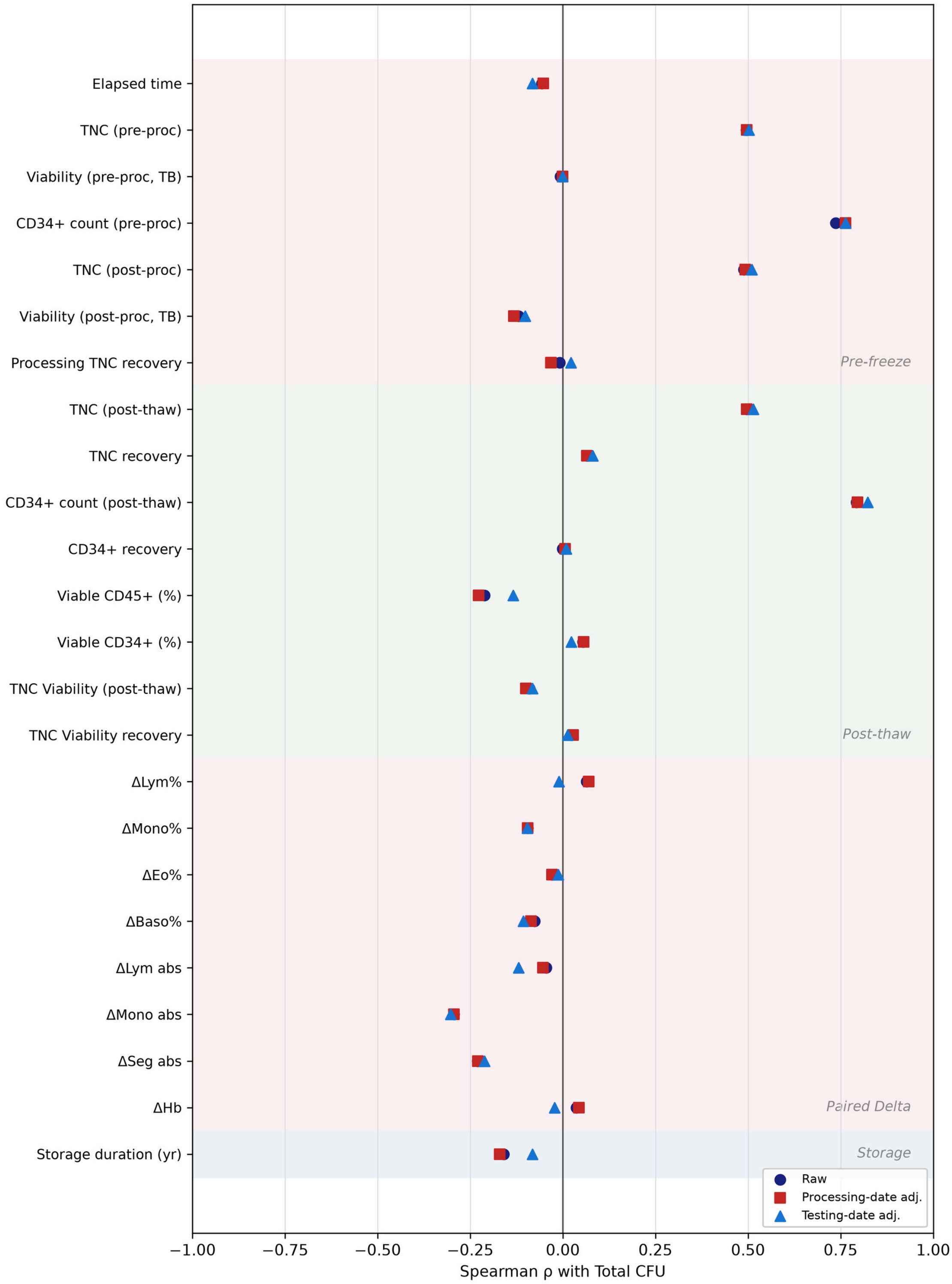

Supplemental Figure S1. Spearman correlation of quality variables with Total CFU: raw vs. era-adjusted (N = 922). Forest-style plot of Spearman ρ for 24 variables under three conditions: raw (dark circles), processing-date adjusted (red squares), and testing-date adjusted (blue triangles). Variables are grouped by category (Pre-freeze, Post-thaw, Paired Delta) and storage duration. CD34-related variables (pre-proc, post-thaw) retain strong positive correlations across all three analyses, whereas storage duration and viable CD45+ show attenuated or reversed signals after testing-date adjustment. Full numerical values are provided in Supplemental Table S1.

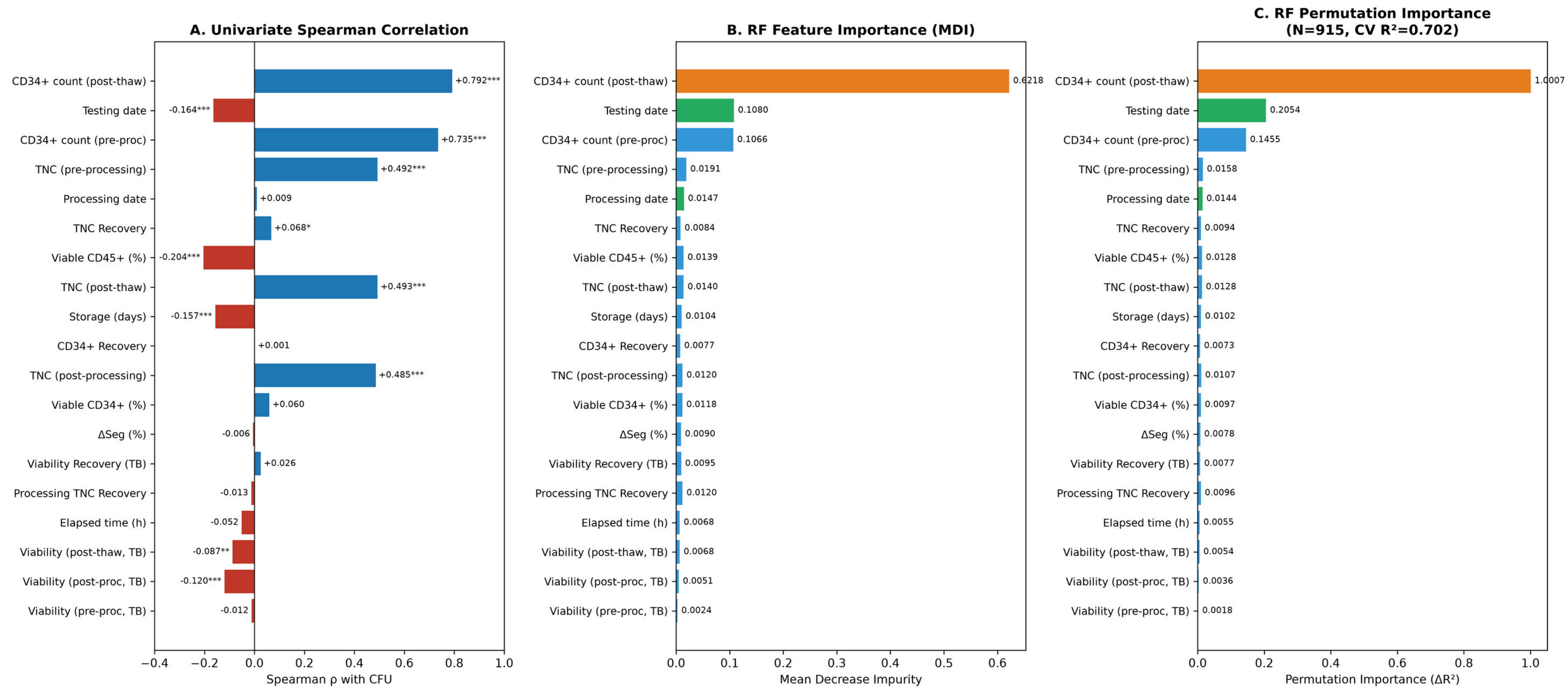

Supplemental Figure S2. Identifying the factors that predict colony-forming capacity (CFU) using Random Forest regression (N = 915; 10-fold cross-validation $R^2 = 0.702 \pm 0.155$). (A) Strength of the simple (univariate) Spearman correlation between each factor and total CFU. (B) Random Forest variable importance by mean decrease in impurity. (C) Random Forest variable importance by permutation (ΔR^2), reflecting each factor's independent contribution once all others are considered together. The simple and multivariate rankings disagree for the two calendar variables: testing date ranks 7th by simple correlation (|ρ|) but rises to 2nd by permutation importance, and processing date rises from 17th to 5th—indicating that these era-related factors act through non-linear and interacting pathways that pairwise correlation cannot detect. Full numerical values are provided in Supplemental Table S2.
